# Validity and reliability of the Brazilian-Portuguese version of three tools to diagnose delirium: CAM-ICU, CAM-ICU Flowsheet and ICDSC

**DOI:** 10.1186/cc10198

**Published:** 2011-06-22

**Authors:** D Gusmao-Flores, JI Salluh, F dal-Pizzol, LR Santana, RM Lins, PP Lemos, GV Serpa, J Oliveira, RA Chalhub, MA Lima, MT Pitrowsky, LC Quarantini

**Affiliations:** 1 Hospital Universitário Prof. Edgar Santos, Universidade Federal da Bahia, Salvador - BA, Brazil; 2 D'Or Institute of Research and Education, Rio de Janeiro - RJ, Brazil; 3 Instituto Nacional do Câncer, Rio de Janeiro - RJ, Brazil; 4 Instituto Nacional de Ciência e Tecnologia Translacional em Medicina, Universidade do Extremo Sul Catarinense, Criciúma - SC, Brazil; 5 Instituto de Ciências da Saúde, Universidade Federal da Bahia, Salvador - BA, Brazil

## Introduction

Delirium is a frequent form of acute brain dysfunction in critically ill patients. Several detection methods have been developed for use in these patients. This study has the objective to validate the Brazilian-Portuguese CAM-ICU and to compare the sensitivity and specificity of three diagnostic tools (ICDSC, CAM-ICU and CAM-ICU Flowsheet) for delirium in a mixed population of critically ill patients.

## Methods

The study was conducted between July and November 2010 in four intensive care units (ICUs) in Brazil. Patients were screened for delirium by a psychiatrist or neurologist as the reference rater using the Diagnostic and Statistical Manual of Mental Diseases, Fourth Edition (DSM-IV), and subsequently by an intensivist rater using a Portuguese translation of the CAM-ICU, CAM-ICU Flowsheet and ICDSC (Intensive Care Delirium Screening Checklist).

## Results

One hundred and nineteen patients were evaluated: 38.6% were diagnosed with delirium by the reference rater. The CAM-ICU had sensitivities of 72.5% (95% CI = 55.9 to 84.9%) and specificity 96.2% (95% CI = 88.5% to 99.0%), the CAM-ICU Flowsheet had sensitivities of 72.5% (95% CI = 55.9 to 84.9%) and specificity 96.2% (95% CI = 88.5% to 99.0%), and the ICDSC had sensitivities of 96.0% (95% CI = 81.5 to 99.8%) and specificity 72.4% (95% CI = 58.6 to 83.0%). High agreement occurred between CAM-ICU and CAM-ICU Flowsheet (kappa coefficient = 0.96).

## Conclusion

The CAM-ICU Brazilian-Portuguese version is a valid and reliable instrument for the assessment of delirium among critically ill patients. The three instruments CAM-ICU, CAM-ICU Flowsheet and ICDSC are good diagnostic tools in critically ill ICU patients and the CAM-ICU was the most specific. In addition, the CAM-ICU Flowsheet presented an excellent correlation with the CAM-ICU and may be employed in general ICU patients.

